# Cigarette and hookah smoking and their relationship with self-esteem and communication skills among high school students

**DOI:** 10.15171/hpp.2018.31

**Published:** 2018-07-07

**Authors:** Masoumeh Anbarlouei, Parvin Sarbakhsh, Hossein Dadashzadeh, Akbar Ghiasi, Maryam Ataieasl, Abbasali Dorosti, Asghar Mohammadpoorasl

**Affiliations:** ^1^Department of Statistics and Epidemiology, Tabriz University of Medical Sciences, Tabriz, Iran; ^2^Research Center of Psychiatry and Behavioral Sciences, Tabriz University of Medical Sciences, Tabriz, Iran; ^3^Research assistant of Health Services Administration, Department of Health Services Administration, the University of Alabama at Birmingham, USA; ^4^Department of Anesthesiology, Tabriz University of Medical Sciences, Tabriz, Iran; ^5^Health and Environment Research Center, Tabriz University of Medical Sciences, Tabriz, Iran

**Keywords:** Cigarette smoking, Hookah smoking, Water-pipe, Adolescents health, Self-esteem, Communication skills

## Abstract

**Background:** Tobacco smoking is one of the most important public health problems that may be prevented. There is limited information about its relationship with communication skills. Findings on the relationships between self-esteem and cigarette/hookah smoking are inconsistent. The aim of this study was to investigate the relationships between cigarette and hookah smoking,self-esteem and communication skills among a representative sample of high school students.

**Methods:** In this cross-sectional study, a sample of 1321 tenth-grade students (14-17 years) was selected through multi-stage proportionally cluster sampling in Tabriz, 2017. The participants completed a self–administered multiple choice questionnaire including questions about cigarette smoking, hookah smoking, self-esteem, and communication skills.

**Results:** After controlling for potential confounders, the results demonstrated that higher score of self-esteem protects students against being in advanced stages of cigarette smoking (odds ratio [OR] = 0.95, 95% CI: 0.92-0.99, P=0.012). However, there was no significant association between self-esteem score and the hookah smoking. Also, there was no significant association between communication skills score and cigarette and hookah smoking.

**Conclusion:** According to our findings, self-esteem was associated with cigarette smoking, but not with hookah smoking. Furthermore, there was no statistically significant association between communication skills score and cigarette and hookah smoking.

## Introduction

Adolescents smoking is associated with significant health problems and it may increase the risk factors of health problems during adulthood.^1^ In order to reduce the health threat caused by tobacco, the World Health Organization (WHO) has targeted 30% decline in the prevalence of tobacco usage among people older than 15 years until 2025 (the base year was 2010).^2^

According to the WHO, the prevalence of tobacco smoking among Iranian adolescents was 5.9%, which is 7.5% among males and 4.2% among females in 2017.^3^Furthermore, the WHO has reported that the prevalence of tobacco smoking among American adolescents was 13.4% (14.6% males and 12.2% females).^3^ In England, 7% of adolescents (6% males and 8% females) are smoking.^3^

There is an increasing trend of smoking prevalence among Iranian adolescents.^4^ For instance, studies have demonstrated that high school students become smoker faster than other groups.^5-7^ Another study in Iran showed that 14.3% and 2.8% of the never smokers became experimenters and regular smokers respectively and 16.5% of the experimenters became regular smokers.^6^ These findings support preventive measures during and before adolescence. Moreover, other studies have reported the prevalence of hookah smoking among adolescents in different cities. For example, according to the result of research was conducted in Tabriz, 44.9% of the high school students had experienced smoking hookah and 6% of them have continued smoking.^8^ Also, another study reported that 21.6% of the students had smoked hookah at least one time during their lifetime, and 9.7% were hookah smoker during study time.^9^ Furthermore, the results of a meta-analysis of articles published between 2000 to 2014 showed that 23% of the high school students in Iran had experienced hookah smoking.^10^

Numerous studies have determined the low self-esteem and deficiency in life skills, especially skills of resistance against smoking as the predictors of smoking.^11,12^ These factors are very important and there is a strong association between them and tobacco smoking among adolescents.^13,14^ In addition, low self-esteem in adolescents increases their smoking tendency during adulthood as well.^15^ Moreover, there is a positive relationship between low self-esteem at school and drug abuse,^16^ which might be provoked by poor performance at school and low approval of the peers.^17^ Nazarzadeh et al found a significant and negative relationship between self-esteem and smoking stages.^18^ Likewise, Khosravi et al indicated that low self-esteem is associated with progress at smoking stages.^19^ On the other hand, Mohammadpoorasl et al found no significant relationship between self-esteem and progress at smoking stages^20^ and between low self-esteem of adolescence and smoking during adulthood.^15^

Considering the inconsistency and ambiguity in the literature of the relationship between self-esteem and communication skills with cigarette and hookah smoking and, particularly, due to limited studies available in Iran, this study aimed to investigate the relationship between self-esteem and communication skills with stages of cigarette smoking and hookah use among a representative sample of adolescents.

## Materials and Methods

### Participants and procedures

This cross-sectional school-based study is the first phase of an interventional longitudinal study about smoking prevention by improving of self-esteem and communication skills training. We studied a representative sample of tenth-grade students in Tabriz (Northwest of Iran) in February 2017. The reason for limiting the sample to tenth-grade students was the possibility of tracking individuals in the next phases of the longitudinal study. To select the participants, the multi-stage proportionally cluster sampling was used. First, nine high schools were selected randomly among all high schools in Tabriz. Secondly, 44 classes were selected as the clusters by considering gender and major of students. Finally, 1321 students were selected for participation in the study.

Using Stata software and taking into account the *P* = 0.17 (transmission rate from experimenter stage to regular smoking stage^20^ ), *P* = 0.085 (expected transition rate after intervention), α = 0.05, and power = 0.90, the required sample size was estimated equal to 345 in each groups. Taking into account the cluster sampling (design effect =1.3), drop-out and missing data, the minimum sample size estimated as 1086. The recruited sample was 1321, but 39 (3.0%) students did not participate. Therefore, 1282 students completed the questionnaire (response rate = 97%). The mean age of student was 15.49 ± 0.50 years (min: 14 and max: 17). Also, 584 (45.6%) of the participants were male and 698 (54.4%) were female.

By explaining the research objectives and obtaining oral informed consent, the selected students entered into the study. Participants filled a self–administered multiple choice anonym questionnaire in their classrooms. The questionnaire was approved by Ethics Committee of Tabriz University of Medical Sciences and Research Committee of the East Azerbaijan Province Education Organization.

### Measures 

The questionnaire includes questions about cigarette smoking, hookah smoking, self-esteem score, communication skills score, substance abuse, self-injury, general risk taking behavior, attitude towards smoking, socio-economical information, and demographic characteristics.

*Communication skills*

Communication skills were assessed by Quondam communication skills test (Persian version). It includes 34 items and it has been adopted and used recently in Iran.^21^ The questions were 5-point Likert scale, ranging from 1 to 5 and some questions were reversely scored. The potential range of score was 34 to 170 and higher scores indicated better communication skills. Test-retest method with a 16 days interval was used to test the reliability of questionnaire in Persian version of the measure among 56 high school students. The Pearson correlation coefficient of measurement obtained 0.79 and the Cronbach α coefficient for the internal consistency of the measurement was 0.91.

*Self-esteem*

Self-esteem was measured using a Persian version of the Rosenberg 10-item questionnaire.^22^ The answer choices of this Likert scaled questionnaire were “completely agree = 4”, “agree = 3”, “disagree = 2” and “completely disagree = 1”. Half of the questions were “negative” and reversely scored. The potential range of score was from 10 to 40, in which the higher scores indicate greater self-esteem. The Pearson correlation coefficient of self-esteem of 56 students with a 16 days interval was 0.79 and the Cronbach α coefficient for the internal consistency of the measurement was 0.88.

*Tobacco use behaviors*

Cigarette smoking status was assessed by a valid algorithm for adolescents’ smoking.^23^ Following the previous studies,^20,24^ cigarette smoking status was considered in 3 stages:

1. Never smoker: students who have never tried smoking of cigarette.

2. Experimenter: students who had tried or smoked less than 100 cigarettes.

3. Regular smoker: students who have smoked 100 cigarettes or more in their lifetime.

Hookah smoking was measured using a multiple choice question with 5 choices including never use, only tried, occasionally use, at least once a month, and at least once a week. Then, the students were categorized in 3 groups of hookah smoking as following:

1. Never smoker: students who have never smoked hookah (even a puff).

2. Experimenter: students who have tried hookah smoking or have smoked occasionally.

3. Regular hookah smoker: Students who use hookah at least once a month.

General risk taking behavior was measured by following question: “Do you enjoy doing things that are a little dangerous or risky?” Respondents with answering “Yes” were classified as having a risky attitude.

Any use of substances such as cannabis, opium, ecstasy and methamphetamines in lifetime was sufficient for students to be classified as “Yes” for substance abuse variable.

Attitude towards cigarette smoking was assessed by a valid and reliable questionnaire which included 6 questions.^20,24^ The potential range of score of this questionnaire was -12 to +12, where higher scores indicated positive attitude.

### Statistical analysis 

Using principal component analysis (PCA) the socioeconomic status was created. Father and mother education, the family assets and the family income were used for PCA. The students were clustered into very high, high, middle, lower and much lower socioeconomic status levels. Because of cluster sampling method, survey analysis was used in all analyses. To analyze data, chi-square test, one-way analysis of variance (ANOVA) and ordinal logistic regression model were used. Stata version 14 (StataCorp., College Station, TX, USA) was used for data analysis.

## Results

Out of all participants, 1038 students (81.5%) were never smoked, 190 (14.9%) experimenter and 45 (3.5%) of them were regular smokers. In terms of hookah smoking, 756 (61.8%), 429 (35.1%) and 38 (3.1%) of students were never smoker, experimenter and regular smoker, respectively.

Table 1 shows the distribution of cigarette smoking by demographic, socioeconomic and behavioral characteristics. As seen in the table, gender, general risk taking behaviors, having self-injury experience, having smoker friend(s), having smoker in the family member(s), hookah smoking status, self-esteem, attitude towards smoking and average grades of previous year were statistically associated with cigarette smoking.

Table 2 shows the distribution of hookah smoking by demographic, socioeconomic, and behavioral characteristics. As shown in this table, gender, general risk taking behaviors, having self-injury experience, having smoker friend(s), having smoker in the family member(s), cigarette smoking status, self-esteem, attitude towards smoking and average grades of previous year were statistically associated with cigarette smoking.

Four ordinal logistic models were used to analysis the relationships between self-esteem and communication skills and cigarette smoking stages and hookah smoking status with and without controlling for potential confounders (Table 3). After controlling for gender, general risk taking behaviors, self-injury experience, substance abuse, smoker friend(s), smoker in the family, hookah smoking status, living with parents, communication skills, attitude towards smoking and average grades of previous year, the results indicated that the higher score of self-esteem protects the students form progressing to advanced stages of cigarette smoking (odds ratio [OR] = 0.95, 95% CI: 0.92-0.99, *P *= 0.012). In fact, for one unit increase in self-esteem score, the odds of higher cigarette smoking stages versus the odds of lower cigarette smoking stages is .95 times smaller, given the other variables are held constant in the model. After controlling for potential confounders, there was no significant association between self-esteem score and hookah smoking status. Also, after controlling for potential confounders, the results did not show any significant association between communication skills score and cigarette smoking stages and hookah smoking status.

## Discussion

The present study is aimed to investigate the relationships between self-esteem and communication skills with stages of cigarette and hookah smoking among adolescents in high schools of Tabriz. The results showed that self-esteem had significant relationship with smoking stages in the sense that each unit of increase in self-esteem score reduces the chance of progressing to the higher stages of smoking by 5%. A study in Shiraz reported an insignificant impact of self-esteem on progressing in smoking stages.^25^ Results of numerous studies have supported the relationship between self-esteem and smoking, or the stages of smoking among adolescents.^18,19,24^ Therefore, it has been suggested that the smoking prevention programs consistently cover the comprehensive health education or health promotion programs such as self-esteem improvement training.^26^ In contrast, another study has shown that self-esteem is a determinant of smoking behavior among female adolescents, but not males, probably because women are exposed to various stimulants for beginning and maintaining the smoking behavior.^27^ However, this study found no significant association between gender and smoking. Based on the results of this study and the literature as well, it seems it is necessary to conduct more studies in this area, especially longitudinal and interventional studies to practically determine the effect of self-esteem improvement programs on smoking prevention.

Regarding to the relationship between self-esteem and stages of hookah smoking, the univariate analysis of variance showed a significant relationship between self-esteem and stages of hookah smoking. In fact, students with monthly smoking of hookah had less self-esteem score than those with experience of hookah smoking; also students with experience of hookah smoking had less self-esteem score than those without experiencing of hookah smoking. However, in the multivariate analysis and by controlling for aforementioned confounders, the relationship was disappeared. Karimy et al have showed that self-esteem is a significant predictor of hookah smoking.^11^ This finding is consistent with the results of univariate analysis in our study. Fakhari et al did not find any significant relationship between self-esteem and hookah smoking.^8^ In spite of the preventive role of family in cigarette smoking families encourage or at least they are neutral about adolescents’ hookah smoking behavior. This can be due to the wrong attitudes about the effect of hookah on health.^28^ Moreover, according to the results of a cross-sectional study conducted in Tabriz on the prevalence of hookah smoking among high school students, 95.3% of regular hookah smokers said that their age is not an impediment for hookah smoking in the coffee shops.^9^ Therefore, it is expectable that due to the accessibility of hookah as well as the lack of legal and social constraints, the smoking of hookah is not considered to be harmful by adolescents. Regarding the role of family, the accessibility of hookah for adolescents in family, as well as considering it as an entertainment in parties, obscenity of hookah smoking seems to be significantly diminished. Therefore, regardless of the status of self-esteem and communication skills, adolescents tend to smoke hookah‏.

There was no significant relationship between communication skills and stages of cigarette smoking. However, Botvin et al found that training the life skills with focusing on increasing the students’ ability to come up with direct pressure of smoking, reducing their sensitivity to indirect social effects of smoking, and improving their ability to reduce anxiety had created a significant difference between intervention and control groups in terms of ratio of the individuals who had experienced tobacco smoking. They concluded that such types of trainings reduce the probability of initiation of smoking behavior in the intervention group.^29^ Consistently, another study confirmed that similar training programs decreased the smoking rate among the trained adolescents compared to control group.^30^ In the other interventional study in 4 states of Germany, the researchers taught students subjects like problem solving, critical thinking, effective communication skills, decision making. The follow-up results in six months after the training showed 16% of the smoker students in the intervention group and 20% in the control group. The difference between two groups was statistically significant.^31^ Results of another interventional study, in which the parents and adults were trained on communication skills, showed that the adolescents of the trained parents began smoking significantly less than others. Therefore, it is suggested that training of the communication skills to adults might affect smoking and other suspicious behaviors of adolescents.^32^ It seems that, finding more reliable and strong results requires conducting further studies, especially longitudinal interventional studies to determine the effect of communication skills training programs on smoking prevention.

The study conducted by Guo et al on the use of training programs including life skills to control and prevent the illegal drug abuse among students showed that the students in intervention group consume significantly less illegal drug than the control group. Also, students in intervention group had more tendency toward avoiding the illegal drugs consumption.^33^ Relying on these findings, it may be assumed that training of communication skills would be effective in preventing hookah smoking. However, this proposition needs to be tested by designing longitudinal interventional study.

## Limitations

This study has several limitations. The main limitation is that the sample size only included tenth grade students. This may reduce the generalizability of the findings. Another limitation was cross-sectional nature of the study that makes it difficult to investigate the causal relationship between independent and dependent variables. Finally, the study relied on self-reported data.

## Conclusion

The findings showed that self-esteem is associated with the stages of cigarette smoking. However, the relationship between self-esteem and hookah smoking was disappeared after controlling for demographic, socioeconomic and behavioral variables. Furthermore, after controlling for potential confounders, the communication skills score showed no statistically significant relationship with both cigarette and hookah smoking. Investigating these relationships requires further studies, especially longitudinal and interventional once to determine the effect of self-esteem improvement programs and communication skills training on the prevention of cigarette and hookah smoking.

## Ethical approval

The research protocol was approved at the organizational committee of ethics in Tabriz University of Medical Sciences (Ethics code: IR.TBZMED.REC.1396.216).

## Competing interests

The authors declare that there is no conflict of interests.

## Authors’ contributions

The first, second and seventh authors contributed to the design of the work. The first and fifth authors conducted the data collection. The first, second and fifth authors conducted the data analyses. The second, third, sixth and seventh authors contributed to the interpretation of the results. The first, fourth, fifth and sixth authors contributed to drafting the manuscript. The sixth and seventh authors supervised the development of work and helped in manuscript evaluation. The fourth author helped to evaluate and edit the manuscript.

## Acknowledgments

The authors would like to greatly acknowledge financial support for this study from Tabriz University of Medical Sciences. They also wish to thank all the participants of this study for their valuable cooperation and participation.

**Table 1:** The distribution of cigarette smoking by demographic and other characteristics of students

**Characteristics**	**Never smoker**	**Experimenter**	**Regular smoker**	**Total**	*P* ** value**
Gender, No. (%)					
Male	440 (75.6)	102 (17.5)	40 (6.9)	582 (45.7)	<0.001
Female	598 (86.5)	88 (12.7)	5 (0.7)	691 (54.3)	
Total	1038 (81.5)	190 (14.9)	45 (3.5)	1273 (100.0)	
Age, No. (%)					
15 years	529 (81.9)	92 (14.2)	25 (3.9)	646 (50.7)	0.655
16 years	509 (81.2)	98 (15.6)	20 (3.2)	627 (49.3)	
Socioeconomic status, No. (%)					
Very low	130 (80.2)	25 (15.4)	7 (4.3)	162 (13.8)	0.846
Low	168 (80.4)	31 (14.8)	10 (4.8)	209 (17.8)	
Middle	191 (78.3)	44 (18.0)	9 (3.7)	244 (20.8)	
High	218 (83.2)	37 (14.1)	7 (2.7)	262 (22.4)	
Very high	244 (82.7)	42 (14.2)	9 (3.1)	295 (25.2)	
Living with parents, No. (%)					
Yes	929 (82.1)	164 (14.5)	39 (3.4)	1132 (92.9)	0.140
No	64 (73.6)	19 (21.8)	4 (4.6)	87 (7.1)	
General risk taking behaviors, No. (%)					
Yes	675 (76.4)	168 (19.0)	41 (4.6)	884 (72.9)	<0.001
No	312 (95.1)	15 (4.6)	1 (0.3)	328 (27.1)	
Having self- injury, No. (%)					
Yes	69 (53.5)	42 (32.6)	18 (14.0)	129 (10.7)	<0.001
No	912 (85.1)	135 (12.6)	25 (2.3)	1072 (89.3)		
Having smoker friend, No. (%)					
Yes	144 (54.3)	86 (32.5)	35 (13.2)	265 (22.1)	<0.001
No	838 (89.4)	95 (10.1)	4 (0.4)	937 (77.9)	
Having smoker in the family, No. (%)					
Yes	292 (74.3)	83 (21.1)	18 (4.6)	393 (32.2)	<0.001
No	702 (84.9)	100 (12.1)	25 (3.0)	827 (67.8)	
Substance abuse, No. (%)					
Yes	3 (50.0)	2 (33.3)	1 (16.7)	6 (0.5)	0.081
No	991 (81.6)	181 (14.9)	42 (3.5)	1214 (99.5)	
Hookah status, No. (%)					
Never hookah smoker	709 (94.0)	35 (4.6)	10 (1.3)	754 (61.9)	<0.001
Hookah experimenter	272 (63.8)	130 (30.5)	24 (5.6)	426 (35.0)	
Regular hookah smoker	12 (31.6)	17 (44.7)	9 (23.7)	38 (3.1)	
Self-esteem, Mean (SD)	32.90 (5.17)	31.12 (5.56)	28.68(6.19)	32.28 (5.44)	<0.001
Communication skills, Mean (SD)	112.04 (10.80)	113.02 (10.27)	110.37(11.26)	112.13 (10.74)	0.290
Attitude toward smoking, Mean (SD)	-10.38 (2.58)	-6.72 (4.07)	-3.86(4.94)	-9.60 (3.41)	<0.001
Previous year average grades, Mean (SD)	18.85 (1.10)	18.38 (1.37)	17.71(1.62)	18.74 (1.20)	<0.001

**Table 2:** The distribution of hookah smoking by demographic and other characteristics of students

**Characteristics**	**Never hookah smoker**	**Experimenter**	**Regular hookah smoker**	***P*** ** value**
Gender, No. (%)				
Male	323 (58.6)	200 (36.3)	28 (5.1)	<0.001
Female	433 (64.4)	229 (34.1)	10 (1.5)	
Total	756 (61.8)	429 (35.1)	38 (3.1)	
Age, No. (%)				
15 years	386 (62.5)	212 (34.3)	20 (3.2)	0.834
16 years	370 (61.2)	217 (35.9)	18 (3.0)	
Socioeconomic status, No. (%)				
Very low	96 (59.3)	64 (39.5)	2 (1.2)	0.062
Low	138 (65.7)	65 (31.0)	7 (3.3)	
Middle	142 (58.2)	97 (39.8)	5 (2.0)	
High	175 (66.3)	77 (29.2)	12 (4.5)	
Very high	170 (57.6)	116 (39.3)	9 (3.1)	
Living with parents, No. (%)				
Yes	708 (62.4)	393 (34.6)	34 (3.0)	0.268
No	47 (54.0)	36 (41.4)	4 (4.6)	
General risk taking behaviors, No. (%)				
Yes	489 (55.1)	361 (40.7)	38 (4.3)	<0.001
No	261 (79.8)	66 (20.2)	0 (0.0)	
Having self-injury, No. (%)				
Yes	48 (37.5)	68 (53.1)	12 (9.4)	<0.001
No	695 (64.6)	356 (33.1)	25 (2.3)	
Having smoker friend, No. (%)				
Yes	107 (40.4)	131 (49.4)	27 (10.2)	<0.001
No	638 (67.9)	291 (31.0)	11 (1.2)	
Having smoker in family, No. (%)				
Yes	197 (50.0)	183 (46.4)	14 (3.6)	<0.001
No	559 (67.4)	246 (29.7)	24 (2.9)	
Substance abuse, No. (%)				
Yes	2 (33.3)	4 (66.7)	0 (0.0)	0.260
No	754 (62.0)	425 (34.9)	38 (3.1)	
Cigarette Smoking status, No. (%)				
Never smoker	709 (71.3)	272 (27.4)	12 (1.2)	<0.001
Experimenter	35 (19.2)	130 (71.4)	17 (9.3)	
Regular smoker	10 (23.3)	24 (55.8)	9 (20.9)	
Self-esteem, Mean (SD)	32.67 (5.43)	31.44 (5.47)	30.32 (6.34)	<0.001
Communication skills, Mean (SD)	111.59 (10.74)	113.10 (10.77)	112.65 (10.07)	0.065
Attitude toward smoking, Mean (SD)	-10.63 (2.48)	-8.09 (3.89)	-6.23 (4.91)	<0.001
Previous year average grades, Mean (SD)	18.86 (1.11)	18.61 (1.25)	18.00 (1.24)	<0.001

**Table 3:** Ordinal logistic regression analysis of the relationship between “cigarette smoking stages” and “hookah smoking status” and “self-esteem” and “Communication skills”

**Variables**	**Cigarette smoking stages**		**Hookah smoking status**	
	**OR (95% CI)**	***P*** ** value**	**OR (95% CI)**	***P*** ** value**
Univariate analysis				
Self-esteem score	0.90 (0.88-0.92)	<0.001	0.95 (0.93-0 .98)	0.012
Communication skills score	1.00 (0.99-1.02)	0.557	1.01 (1.00-1.02)	0.203
Multivariate analysis^a^				
Self-esteem score	0.95 (0.92-0.99)	0.012	1.02 (0.99-1.05)	0.084
Communication skills score	1.01 (0.99-103)	0.404	1.01 (0.99-1.02)	0.113

Abbreviation: OR, odds ratio. ^a^ Adjusted for gender, general risk taking behaviors, self-injury experience, substance abuse, smoker friend(s), smoker in the family, hookah smoking status, living with parents, communication skills (or self-esteem), attitude toward smoking and previous year average grades.
